# A Helicopter Perspective on TB Biomarkers: Pathway and Process Based Analysis of Gene Expression Data Provides New Insight into TB Pathogenesis

**DOI:** 10.1371/journal.pone.0073230

**Published:** 2013-09-16

**Authors:** Simone A. Joosten, Helen A. Fletcher, Tom H. M. Ottenhoff

**Affiliations:** 1 Department of Infectious Diseases, Leiden University Medical Center, Leiden, The Netherlands; 2 Jenner Institute, University of Oxford, Oxford, United Kingdom; University of Palermo, Italy

## Abstract

Biomarker host genetic signatures are considered key tools for improved early diagnosis of tuberculosis (TB) disease (development). The analysis of gene expression changes based on a limited number of genes or single study designs, however, may not be sufficient for the identification of universal diagnostic biomarker profiles. Here we propose that biological pathway and process based analyses from multiple data sets may be more relevant for identification of key pathways in TB pathogenesis, and may reveal novel candidate diagnostic TB biomarkers. A number of independent genome-wide gene expression studies have recently been performed to study expression of biomarkers for TB disease. We have integrated the results from these independent studies and performed pathway- as well as biological process-based analysis on the total data set. Interestingly, IFNα/β signalling is not the single dominant pathway in the analysis of the total dataset, but combined, functional, analysis of biomarkers suggests a strong dominant role for myeloid cell involvement in inflammation.

## Introduction

Biomarkers are defined as: “characteristics that can be objectively measured and evaluated as an indicator of a normal biological process, a pathogenic process, or a (pharmacologic) response to a therapeutic intervention” [1]. Biomarkers could be of great significance in the battle against tuberculosis (TB), especially in the development of better diagnostics and new vaccines [2]. A number of new TB vaccine candidates have been developed over the past decade and many of them are currently in early stage clinical development. The next major step will be to test the protective efficacy of these new candidates, which will present a major challenge because of the nature of TB infection and its progression to disease: in most individuals, infection with *Mycobacterium tuberculosis* (Mtb) results in latent infection that can persist for decades, with active disease development in about 3–10% of infected individuals, mostly within the first 2 years post infection [3], [4]. The low incidence rates of TB disease require large and lengthy clinical vaccine efficacy trials in TB endemic areas to achieve the statistical power needed to demonstrate vaccine induced protection against disease development.

As an alternative to using clinical endpoints, scientists are urgently searching for biomarkers that predict whether individuals are (long-term) protected or at (increased) risk for disease development. Such biomarkers would allow assessment of vaccine efficacy at earlier stages and with smaller groups of individuals, and allow comparison of multiple TB vaccine candidates in efficacy studies. Thus, the demonstration of TB vaccine efficacy and biomarker efficacy are both of eminent importance. Such biomarkers would also facilitate licensure of new efficacious TB vaccines in different age groups (infants, children, adults) as well as ethnically and geographically different populations (particularly Africa and Asia) without having to perform further large scale efficacy testing.

### Biomarker Challenges

Identification of biomarkers of protection against TB disease is challenging because there is no gold standard of infection or protection. Our understanding of what is strictly required for host protection from TB disease is incomplete, which hampers vaccine development [2], [2,5]. Moreover, validation of biomarkers against clinical endpoints is difficult because in TB clinical endpoints cannot always be clearly defined. For the curative response to treatment time to sputum conversion is frequently used, but for protection against disease development no gold standard clinical definition is available. In infant BCG vaccination studies, protection has been defined as known exposure to TB within the household without the development of disease within 2 years following exposure [6]. This definition is probably suitable for infants that experience most exposure within the household, however, for most (adult) people it is difficult to trace exposure to TB and thus disease-free time post exposure [7]; moreover, adults may more often be re-exposed during the 2 year follow up period, confounding estimations of disease incidence. Clinical endpoints should be comparable among studies to allow ranking and prioritization of biomarkers [2]. An additional complexity is that new candidate TB vaccines are quite different by nature, *e.g.* live vaccines compared to subunit vaccines or priming compared to boosting vaccines, which each may induce protection in a different manner and may thus require different sets of biomarkers to evaluate vaccine induced protection [2].

Next to biomarkers of protection against TB disease development, also biomarkers predicting disease risk are extremely important in vaccine trials: these may identify individuals at risk at an early stage and thus help to significantly shorten follow-up times and numbers. Biomarkers of early TB disease development may be easier to identify since in contrast to protection, TB disease can be demonstrated using microbiological diagnosis (bacterial staining or culture, GeneXpert MTB/RIF), X-ray or made highly likely based on clinical manifestations. To identify biomarkers of disease-risk, patients can be compared to various other groups, including those with latent TB, treated TB patients or patients with other inflammatory or infectious diseases. Early identification of individuals at risk of developing TB disease will help reducing the occurrence of contagious pulmonary TB and thus limit disease transmission.

Biomarkers will thus be critical tools in the battle against TB. However, a number of issues need to be taken into consideration when comparing biomarker studies. First, geographic or ethnic variations may significantly impact on immune responses towards TB due to a variety of factors which include: host genetic factors; the presence of environmental microbes like helminths or HIV [8], [9] (Table 1); exposure to/infection with immunomodulatory (environmental) non-tuberculous mycobacteria; previous BCG vaccination; *Mtb* exposure intensity and frequency; reinfection rates; *Mtb* strain heterogeneity etc. Concomitant HIV infection may predominantly affect biomarkers derived from the CD4^+^ T cell compartment. In addition, metabolic conditions may influence immunity and biomarker signatures following vaccination or infection. For example, obesity and type 2 diabetes mellitus (T2D) both result in continuous low-grade systemic inflammation, including cytokine and chemokine production, switching of macrophage subsets from anti-inflammatory Mf2 to pro-inflammatory Mf1 [10], [11]. The significance of T2D for TB has emerged from epidemiological data and prompted WHO to initiate combined care for TB and T2D [12]–[15]. Patients with T2D have a 3-fold increased risk to develop TB disease, and achievement of negative sputum cultures as a measure of treatment success takes longer as compared to non-T2D TB patients [12]–[15]. Also low body mass indexes and malnutrition are significant risk factors for the development of TB disease, indicating that nutritional status is an important factor for TB disease, which may significantly affect measurable biomarker profiles [14], [16].

**Table 1:** Factors that may affect TB Biomarkers.

patient	age				
	geographic origin/ethnicity				
	environmental exposure (eg environmental mycobacteria)				
	previous vaccinations (eg BCG during childhood)				
	co-infections (eg HIV, Helminths)				
	metabolic state (malnutrition, obesity, T2D)				
	use of immunomodulating drugs (eg immune suppression)				
pathogen	strain (eg MDR, Beijing)				
	route of entrance (eg vaccine vs natural infection)				
	site of disease (pulmonary vs extrapulmonary)				
	time since infection/stage of disease progression				
sample	type of sample (eg whole blood, PBMCs, fluid from disease site)				
	time between collection and fixation				
	sample handling (eg isolation procedures, temperature)				

Secondly, the type of material used for biomarker determination may greatly determine the type of biomarkers that can be detected. Whole blood contains large numbers of neutrophils, which are present at a very low frequency in isolated PBMCs, and which seem to be cells that express promising TB biomarkers [17]–[20]. On the other hand, the strong signal from these large numbers of neutrophils in whole blood may obscure highly relevant and specific gene expression profiles from smaller populations, e.g. T-cells, B-cells, monocytes or other relatively rare populations. Furthermore, it is recognized that the isolation of cells from whole blood may alter their gene expression profiles, which is further enhanced if blood processing is delayed for several hours [21]–[23]. Therefore, biomarkers should preferably be analysed on the same type of material over different studies and processing time should be standardized to reduce variation (Table 1) [22]–[25]. These differences in cell populations may be reflected in differential expression of particular biomarkers, and thus not only reflect the response to TB but also indicate changes in comorbidities. Finally, biomarkers for different forms of disease may be different, e.g. pulmonary vs. extrapulmonary TB, partly because samples obtained from peripheral blood, sputum or pleural fluid have different cellular compositions.

### TB biomarkers and other diseases

The use of biomarkers to predict TB disease progression (or ultimately vaccine efficacy or protection) is complicated by the nature of these markers. Most TB biomarkers or biomarker-signatures identified so far are indicators of general (intracellular) infection and subsequent immune activation, rather than highly specific for TB disease. There is a large overlap with biomarkers reported in other inflammatory diseases including SLE, sarcoidosis, melioidosis and Still's disease [17], [26], [27], as well as following Yellow Fever vaccination [28]. With the exception of sarcoidosis and melioidosis, most of these diseases can be discriminated rather easily from TB based on clinical symptoms or examination in combination with existing diagnostics.

It is unsurprising that TB biomarker profiles overlap significantly with other infections and inflammatory conditions, since most pathways represent genes associated with immune activation and inflammation. At the cellular level, infection with any pathogen may trigger activation of innate cells through pattern recognition receptors (TLRs, NLRs etc.), resulting in upregulation of markers and cytokines/chemokines irrespective of the exact nature of the pathogen. Following specific recognition by the adaptive immune system, this will similarly result in activation of innate cells, initiating a modular response to eliminate the invading pathogen. Thus, it is to be anticipated that a large number of biomarkers should be shared between disease processes that rely on similar host-module responses.

### Global TB Biomarker Signatures

Multiple studies have analysed production of single cytokines or chemokines, or the expression of cell surface markers at the protein or mRNA level as biomarkers for TB disease. More recently, single candidate biomarkers were combined into multicomponent signatures to increase power and specificity. Advanced statistical methods were applied to select genes from (global) transcriptomic datasets and to compile signatures including the smallest possible number of genes to retain good predictive values. Such signatures appear to be more powerful biomarkers than individual genes or proteins [19], [29], [30].

Recently, multiple groups have reported global gene expression analysis in different cohorts of TB patients with active disease (Table 2). In these studies, signaling through the type I interferon (Interferon alpha/beta (IFNα/β)) pathway was frequently reported as important in TB disease, although the molecules identified as differentially expressed or produced were not identical between all different cohorts or studies. Because the pathways involved are clearly related and overlapping, we decided to analyse the TB biomarker signatures emerging from these genome wide transcriptomic studies as a group, using a “helicopter” perspective by jointly analyzing data from all published genome wide expression studies on TB disease to date. A helicopter view over all data may allow comparative analysis of all available data rather than a comparison of individual pathways identified by each study. Since all data are assessed to be equally important the distant view from the helicopter should allow identification of major players in the TB disease development. This should help to better characterize the key processes and pathways involved rather than identifying markers which might be relatively unique to certain study settings, determined e.g. by the specific population sampled, time and type of sampling, and other possible confounders discussed above. We reasoned that the processes or pathways identified by such an analysis should be more reliable and generic, and thus may provide a platform for further TB biomarker exploration and evaluation in a global context.

**Table 2:** Information on studies included.

study	reference	population	sample collection	# TB patients	type of sample
				initial, independent	
1	Mistry R, JID, 2007: 195, 357	active TB disease vs healthy infected controls	South Africa	n = 10	whole blood
2	Jacobsen M, J Mol Med, 2007: 85, 613	active TB disease vs healthy infected controls	Germany	n = 9	PBMC
3	Berry MP, Nature, 2010: 466, 973	active TB disease vs healthy infected & uninfected controls vs other inflammatory disorders (SLE, Stills, Streptococcus, Staphylococcus)	United Kingdom (test), South Africa (validation)	n = 13, n = 20	whole blood
4	Maertzdorf J, Genes & Immunity, 2011: 12, 15	active TB disease vs healthy infected & uninfected controls	South Africa	n = 33	whole blood
5	Maertzdorf J, PLoS ONE, 2011: 6, e26938	active TB disease vs healthy infected & uninfected controls	The Gambia	n = 46	whole blood
6	Maertzdorf J, PNAS, 2012: 109, 7853	active TB disease vs heatlhy infected & uninfected controls & sarcoidosis	Germany	n = 8	whole blood
7	Cliff J, JID, 2013: 207, 18	active TB disease over time during treatment	South Africa	n = 27, n = 9	whole blood
8	Ottenhoff TH, PLOS ONE, 2012: 7, e45839	active TB disease over time during treatment vs healthy controls	Indonesia	n = 23	PBMC

## Methods

### Data input

Over 6 years eight independent genome wide expression studies have been performed on blood from patients with active TB disease (Table 2) [17]–[20], [26], [29], [31], [32]. Individuals with active TB disease were compared to different control populations, including: patients longitudinally followed during treatment; healthy (infected) individuals; and patients with other (infectious) diseases (Table 2). Patients originated from different geographical regions, including Europe, Asia, Saharan and Sub-Saharan Africa. Gene expression data were analysed within each study, mostly involving pathway analysis to determine the most dominant signaling pathways within that cohort in comparison to their specific control population. Gene signatures were determined that could serve as biomarkers to discriminate patients with active TB disease from the respective control population included. A number of studies also included pathway ontology analysis to decipher the potential cell subsets or cellular processes that were most different between TB disease and control populations.

Here, we combined all genes identified by each of the eight independent global gene expression studies to discriminate between patients with active TB disease and controls, into a single data set, allowing more comprehensive analysis of gene expression changes during TB disease. All genes identified by each of the individual studies as differentially expressed between TB disease and their respective control populations were included in this analysis. Gating criteria for gene selection were determined by the individual authors and their selection of genes differentially expressed in TB disease versus their respective control population was added into our study, irrespective whether the genes were up or downregulated. A total gene-set of 409 genes was the result of combining all 8 individual studies.

### Analysis

**Figure 1:**
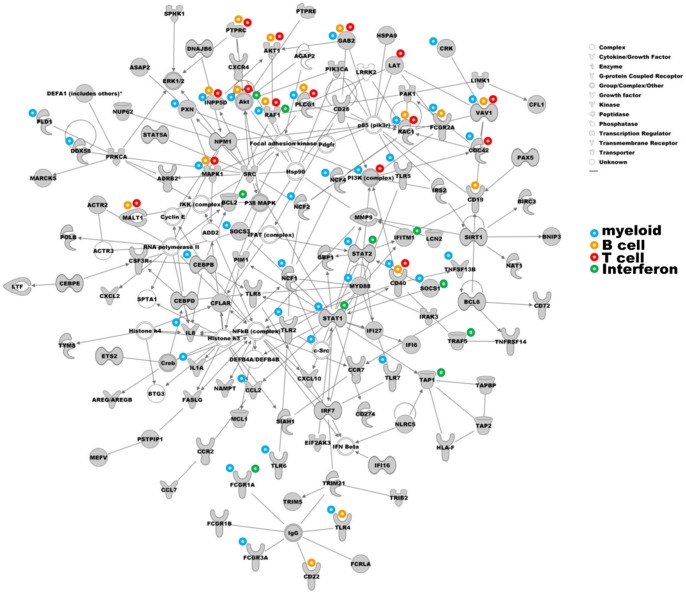
Ingenuity pathway analysis of all genes identified by unbiased methods related to TB disease. All 409 biomarkers were analysed by integrated pathway analysis using Ingenuity and the most dominant network is depicted here. Signalling pathways were coloured according to functional classification into myeloid cells, T cells and B cells and type I interferon related genes.

The total gene-set (409 genes) from all studies mentioned in Table 2 was analysed functionally using 3 different platforms:

- Modular analysis: grouping based on similar expression kinetics over multiple diseases/processes as described by Chaussabel et al [33]. We have used the updated version of the modules that is available at: http://www.biir.net/public_wikis/module_annotation/G2_Trial_8_Modules. Genes (both formal gene names according to genecards.org and alternative names as specified in Table S1) were searched within the modules and indicated in Table S1. Colour coding was performed based on the name of the modules and thus all modules named ‘interferon’ or ‘inflammation’ received the same colour.
- Molecular interactions: association of genes into ‘integrated pathway analysis’ (IPA), including analysis of canonical pathways involved using Ingenuity™ platforms, available at: www.ingenuity.com. All 409 genes were inserted into Ingenuity integrated pathway analysis and network generation was performed to search for defined molecular interactions between genes (gene products). Results were visualized as networks (Figure 1) and ranked as canonical pathways involved (Table 3).
- Biological pathways: Gene Set Enrichment Analysis (GSEA) [34]. Genes were tested against the ‘Molecular signature Database’ (MsigDB, http://www.broad.mit.edu/gsea/msigdb) C2 collection (4850 gene sets). All 409 genes were compared to the database of 4850 known datasets to search for the datasets with the highest overlap in gene-expression profiles and thereby to identify processes or diseases that mimic our set of genes derived from TB disease patients (Table 4).

**Table 3:** Results of Ingenuity pathway analysis.

Ingenuity Canonical Pathways		-log (p-value)	p-value	Ratio Overlap with dataset	
	categorie in table S1		(approximate)		
TREM1 Signaling	TREM 1 signalling	1,6E01	1,00E-16	3,33E-01	19/57 (33%)
Fcγ Rec.-mediated Phagocytosis in Macroph. and Monoc.	myeloid cells	1,46E01	2,50E-15	2,32E-01	22/95 (23%)
Mitochondrial Dysfunction	mitochondria	1,39E01	1,12E-14	1,85E-01	25/135 (19%)
Pattern Recognition Rec. in Recognition of Bacteria and Viruses	myeloid cells	1,37E01	1,99E-14	2,21E-01	21/95 (22%)
Macroph., Fibrobl. and Endothelial Cells in Rheumatoid Arthritis	myeloid cells	1,18E01	1,58E-12	1,06E-01	33/311 (11%)
B Cell Receptor Signaling	B cell	9,78E00	1,65E-10	1,36E-01	22/162 (14%)
PI3K Signaling in B Lymphocytes	B cell	9,21E00	6,16E-10	1,48E-01	19/128 (15%)
Communication between Innate and Adaptive Immune Cells	–	8,43E00	3,70E-09	1,61E-01	15/93 (16%)
Dendritic Cell Maturation	myeloid cells	8,3E00	5,00E-09	1,09E-01	21/192 (11%)
Systemic Lupus Erythematosus Signaling	inflammation	8,17E00	6,76E-09	1,01E-01	23/228 (10%)
IL-8 Signaling	inflammation	7,72E00	1,90E-08	1,09E-01	21/192 (11%)
Natural Killer Cell Signaling	–	6,76E00	1,73E-07	1,36E-01	15/110 (14%)
Prod. of Nitric Oxide and Reactive Oxygen Species in Macroph.	myeloid cells	6,5E00	3,16E-07	1,02E-01	19/186 (10%)
NF-κB Signaling	inflammation	6,45E00	3,50E-07	1,06E-01	18/170 (11%)
Role of Tissue Factor in Cancer	–	6,03E00	9,33E-07	1,28E-01	14/109 (13%)
Erythropoietin Signaling	hematopoiesis	5,85E00	1,40E-06	1,49E-01	11/74 (15%)
Altered T Cell and B Cell Signaling in Rheumatoid Arthritis	inflammation	5,83E00	1,47E-06	1,4E-01	12/86 (14%)
Toll-like Receptor Signaling	myeloid cells	5,79E00	1,62E-06	1,75E-01	10/57 (18%)
IL-3 Signaling	hematopoiesis	5,59E00	2,57E-06	1,51E-01	11/73 (15%)
CD28 Signaling in T Helper Cells	T cells	5,01E00	9,77E-06	1,07E-01	13/122 (11%)
IL-12 Signaling and Production in Macrophages	myeloid cells	4,98E00	1,05E-05	1,02E-01	14/137 (10%)
Chemokine Signaling	inflammation	4,92E00	1,20E-05	1,47E-01	10/68 (15%)
FAK Signaling	–	4,76E00	1,74E-05	1,12E-01	11/98 (11%)
FLT3 Signaling in Hematopoietic Progenitor Cells	hematopoiesis	4,75E00	1,78E-05	1,37E-01	10/73 (14%)
Atherosclerosis Signaling	–	4,73E00	1,86E-05	9,92E-02	13/131 (10%)
iCOS-iCOSL Signaling in T Helper Cells	T cells	4,73E00	1,86E-05	1,07E-01	12/112 (11%)
Rac Signaling	–	4,73E00	1,86E-05	1,03E-01	12/117 (10%)
Apoptosis Signaling	–	4,67E00	2,14E-05	1,2E-01	11/92 (12%)
Prolactin Signaling	hematopoiesis	4,65E00	2,24E-05	1,3E-01	10/77 (13%)
Pancreatic Adenocarcinoma Signaling	–	4,6E00	2,51E-05	1,04E-01	12/115 (10%)
PDGF Signaling	–	4,44E00	3,63E-05	1,27E-01	10/79 (13%)
IL-6 Signaling	inflammation	4,21E00	6,17E-05	9,84E-02	12/122 (10%)
HGF Signaling	hematopoiesis	4,19E00	6,45E-05	1,08E-01	11/102 (11%)
G Beta Gamma Signaling	–	3,94E00	1,15E-04	1E-01	10/100 (10%)
Fc Epsilon RI Signaling	–	3,81E00	1,54E-04	9,91E-02	11/111 (10%)
T Cell Receptor Signaling	T cells	3,62E00	2,39E-04	9,8E-02	10/102 (10%)
IGF-1 Signaling	–	3,59E00	2,57E-04	9,8E-02	10/102 (10%)

All canonical pathways significantly associated with the dataset are depicted (p<0.001), after application of the following filter criteria: gene set comprises at least 50 genes, at least 10 genes from dataset are retrieved in gene set and at least 10% of genes from gene set are present in the data set of 409 genes.

**Table 4:** Results from GSEA.

GSEA gene expression data sets	categorie in table S1	NES	FDR q-val	SIZE
REACTOME_PEPTIDE_LIGAND_BINDING_RECEPTORS	myeloid cells	2,07	0,041	15
REACTOME_GPCR_LIGAND_BINDING	myeloid cells	2,08	0,041	18
SEKI_INFLAMMATORY_RESPONSE_LPS_UP	inflammation	2,14	0,034	18
SMID_BREAST_CANCER_NORMAL_LIKE_UP	inflammation	2,15	0,034	31
PICCALUGA_ANGIOIMMUNOBLASTIC_LYMPHOMA_UP	T cells	2,32	0,041	18
MOSERLE_IFNA_RESPONSE	interferon	2,25	0,028	16
LIU_VAV3_PROSTATE_CARCINOGENESIS_UP	inflammation	2,16	0,037	16
SENGUPTA_NASOPHARYNGEAL_CARCINOMA_WITH_LMP1_UP	inflammation	2,12	0,032	17
SEITZ_NEOPLASTIC_TRANSFORMATION_BY_8P_DELETION_UP	myeloid cells	2,08	0,04	15
ICHIBA_GRAFT_VERSUS_HOST_DISEASE_35D_UP	inflammation	2,13	0,033	25
ICHIBA_GRAFT_VERSUS_HOST_DISEASE_D7_UP	inflammation	2,17	0,037	26
TAKEDA_TARGETS_OF_NUP98_HOXA9_FUSION_3D_UP	myeloid cells	2,27	0,04	26
MARKEY_RB1_ACUTE_LOF_UP	inflammation	2,52	0,014	39
JISON_SICKLE_CELL_DISEASE_UP	inflammation	2,25	0,023	36
REACTOME_IMMUNE_SYSTEM	inflammation	2,25	0,034	101

Gene sets with an FDR <5% were included in this analysis. SIZE indicates the number of genes in both the gene set and the expression dataset. NES the primary result of the gene set enrichment analysis is the enrichment score (ES), which reflects the degree to which a gene set is overrepresented in a list of genes. Normalizing the enrichment score (NES) accounts for differences in gene set size and in correlations between gene sets and the expression dataset.

## Results and Discussion

### Pathway analysis of state-of-the art Biomarker data

**Figure 3:**
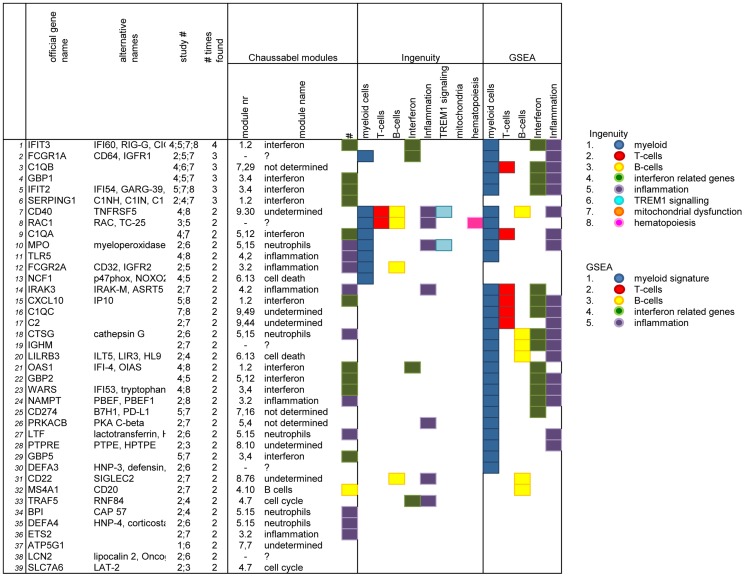
Genes identified by more than 1 independent study. Genes identified by more than 1 independent global genome-wide gene expression analysis. Manuscript numbers refer to Table 2. Classification into modules, functional groups according to Ingenuity and GSEA was performed according to Tables 3 & 4 and identical to Genes identified by more than 1 independent global genome-wide gene expression analysis. Manuscript numbers refer to Table 2. Classification into modules, functional groups according to Ingenuity and GSEA was performed according to Tables 3 & 4 and identical to Table S1.

The combination of individual markers from these 8 independent studies (table 2) [17]–[20], [26], [29], [31], [32], yielded 409 genes associated with TB disease, 39 of which were identified by more than 1 independent study. All 409 genes are summarized in Table S1 and include a reference to the original studies that have identified these genes as well as the number of studies that identified that particular gene. All genes identified by more than one study are separately analysed in Figure 3.

#### Modular analysis

**Figure 2:**
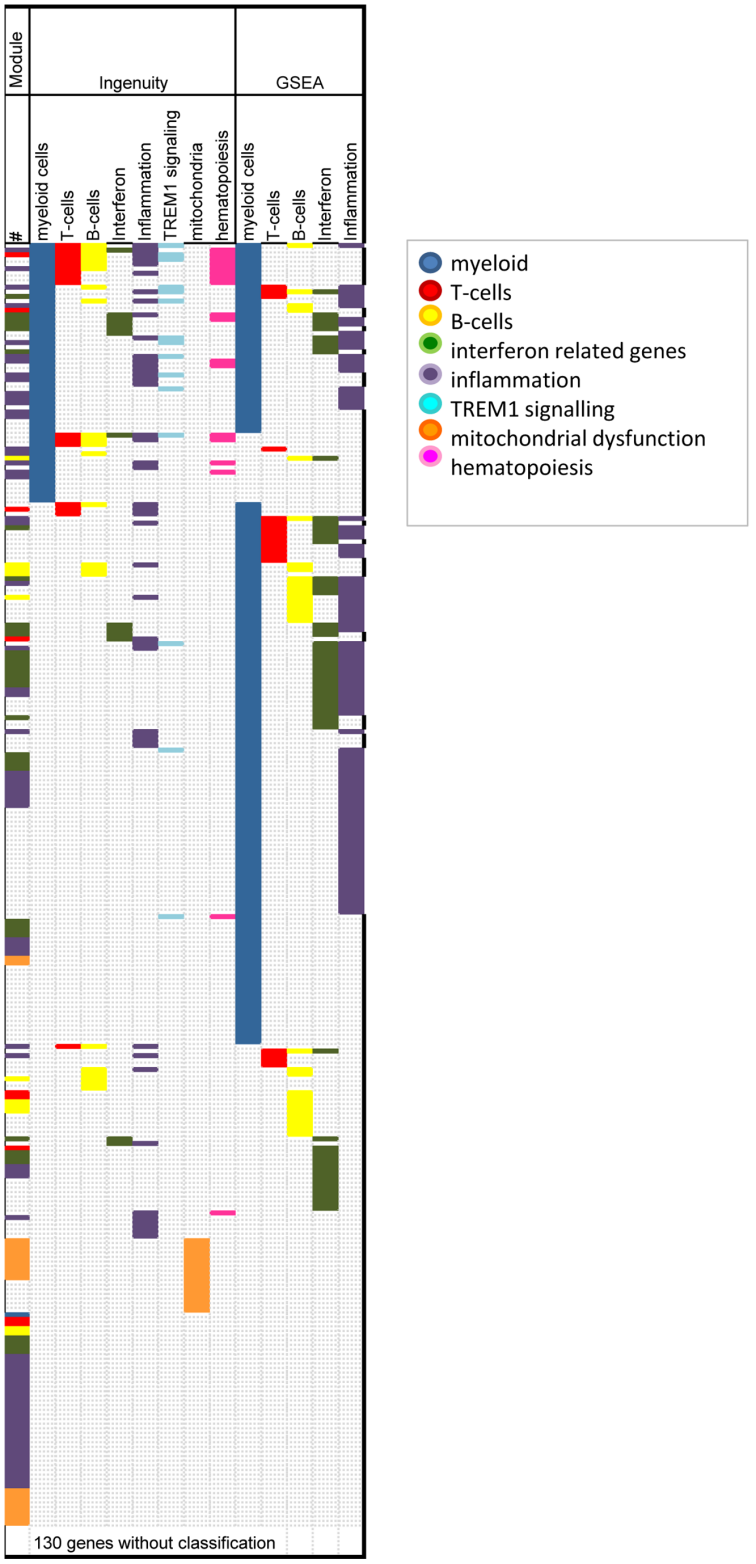
Functional classification of individual genes identified by gene expression analysis on TB patients. Categories have been based on combined output from Ingenuity and GSEA software modules and may include multiple canonical pathways or cell processes. Myeloid cells includes the following canonical pathways: role of macrophages, fibroblasts and endothelial cells in rheumatoid arthritis; Fcg Receptor mediated phagocytosis in macrophages and monocytes; role of pattern recognition receptors in recognition of bacteria and viruses; IL12 signaling and production in macrophages; Dendritic cell maturation; production of Nitrox Oxide and Reactive Oxygen Species in Macrophages; Toll like receptor signaling. T cells includes: T cell receptor signaling; CD28 signaling in T helper cells; iCOS-iCOSL signaling in T helper cells. B cells includes: B cell receptor signaling; PI3K signaling in B lymphocytes. Interferon related pathways include: Interferon signaling, role of jak1, jak2 and tyk2 in interferon signaling, role of PKR in interferon induction and antiviral response. Inflammation includes: IL-8 signaling; NF-kB signaling; altered T cell and B cell signaling in Rheumatoid Arthritis; systemic lupus erythematosus signaling; chemokine signaling; IL-6 signaling. TREM1 includes specifically TREM1 signaling and mitochondrial dysfunction also only contains mitochondrial dysfunction. Finally, hematopoiesis includes: erythropoietin signaling; IL-3 signaling; FLT3 signaling in hematopoietic progenitor cells; prolactin signaling; HGF signaling.

Categorizing individual genes is a useful tool to obtain insights in relative representations of functional groups in the dataset. A number of independent studies have used the modular classification described by Damian Chaussabel [33], which is based on the assumption that the probability for multiple transcripts to follow a complex pattern of expression across dozens of conditions only by chance is low and such sets of genes should therefore constitute coherent and biologically meaningful transcriptional units. He used microarray based gene expression profiles from a number of diseases and grouped genes according to their concordance in expression profiles [33]. Pubmed searches were done to identify module functional associations, resulting in rather broad, cell type based allocations in particular as a consequence of using PBMCs as source material. Conceptually the modular framework is very elegant and expected to give insights in processes that are strongly enriched within a gene expression data set. However, the number of genes that can be grouped into the modules initially was limited. Recently, a novel version of these modules was published, which is now based on Illumina microarray platforms and contains a larger number of genes (publically available at: http://www.biir.net/public_wikis/module_annotation/G2_Trial_8_Modules). Also the number of modules was greatly expanded in this new version compared to the previous version [33]. All 409 genes in our current data set were annotated according to the latest version of the modules (web publication date August 2012) (Table S1). 371/409 (90.7%) genes in the combined dataset were retrieved and assigned to modules (Table S1). However, the majority of genes (195/371 or 52.6%) were allocated to modules without functional assignment (undetermined/not determined), resulting in only 176/409 (43.0%) of genes with a functional classification (Table S1, Figure 2). Two modules are highly represented in the dataset: 68/176 (36.6%) of the allocated genes belong to the inflammatory signature and 36/176 (20.5%) genes belong to the interferon related signature. Genes identified in more than 1 independent studies also predominantly represented patterns of inflammation (9/39; 23%) and interferon related pathways (7/39; 17.9%) (Figure 3).

The modular analysis as described above thus hints towards a prominent role for inflammation and interferon signaling in TB disease, as was also described by most individual studies. The disadvantage of the modular data analysis is the limited relation of gene sets within a module to specific functional capacities, the relative lack of sensitivity for small changes in gene expression profiles, mostly because the data sets used to annotate the modules were all derived from PBMCs, with their particular cellular composition, and did not include specialized cell types, or specifically activated pathways. For example, monocytes and macrophages only represent a low proportion of PBMCs (5–10%) and thus specific monocyte activation pathways may not be recognized by the modular annotation.

#### Molecular Interactions

Ingenuity based pathway analysis revealed highly significant overlap with 92 canonical pathways with p<0.001 and a total of 196 canonical pathways p<0.05. To select the most important pathways within our dataset we defined and applied filter criteria on the 92 canonical pathways with the highest significance. First, pathways were excluded if they were smaller than 50 genes; secondly, pathways were excluded if fewer than 10 out of the total of 409 gene dataset mapped in that pathway; and thirdly, we only considered pathways in which our genes represented at least 10% of the total gene-set in that pathway. This resulted in 36 pathways that were significantly abundant in our data set of 409 genes (Table 3, Figure 2).

#### Biological pathways

Gene Set Enrichment Analysis (GSEA) [34] determines whether there is significant overlap or enrichment of genes in published biological pathways with genes in a query list. To evaluate the degree of enrichment the GSEA method calculates an Enrichment score (ES) and False Discovery Rate (FDR). We tested our set of 409 genes against the Molecular signature Database C2 collection (4850 gene sets) collected from various sources such as online pathway databases, publications in PubMed, and knowledge of domain experts. In the GSEA analysis 230 gene sets were returned with a significant enrichment score (ES) although only 15 of these gene sets were significant at an FDR of <5% (Table 4, Figure 2).

### Pathway and process based analysis

#### TREM1 signaling pathway

The Ingenuity canonical pathway overlapping the combined dataset with highest significance level was ‘TREM1 signaling’ (Table 3). Genes that comprise the TREM1 canonical pathway are depicted in Table 5, in addition genes identified in our dataset are indicated. The dataset overlaps with TREM1 signaling directly, but also has significant overlap with TLR and downstream TLR signalling as well as with effector cytokines secreted following TREM1 activation. This is a novel finding, not detected in any of the individual published studies. TREM1 or ‘Triggering receptor expressed on myeloid cells 1’ is a member of the Ig superfamily and is predominantly expressed on myeloid cells. Cell surface expression of TREM1 is increased upon cellular activation, e.g. by LPS and other microbial products [35]. Expression of TREM1 results in amplification of neutrophil- and monocyte-mediated inflammatory responses, by increased cytokine production and upregulation of cell activation markers, as seen in bacterial and fungal infections. TREM1 signaling pathways interact with signaling pathways downstream from the IFNaR1/2 and from TLRs [36], [37]. Cross-talk between TREM1 and TLR2 and TLR4 signaling cascades have been demonstrated, and potentially also other TLR (TLR 3, 5, 7, 8, and 9) and possibly NLR signaling pathways are influenced by TREM1 signaling [37]. TREM1 and TLR2 signaling pathways can synergize at the level of cytokine production [37]. TREM1 plays a crucial role in fine-tuning of the inflammatory response by amplifying or dampening TLR induced signals, and is known to tune the septic response in order to facilitate efficient clearance of the pathogen without damaging the host [36]. The ligand of TREM1 is currently unknown, although it has been speculated that it may be expressed on pathogens, thereby directly activating TREM1 signaling [37]. Peptidoglycans on gram positive bacteria and endotoxins on gram-negative bacteria are candidate ligands, and mycobacterial peptidoglycans might also serve as TREM1 ligands. This remains to be determined but would be an interesting explanation for the increased TREM1 signaling observed in patients with active TB disease.

**Table 5:** TREM1 canonical pathway.

	identified in study nr:
Akt	5
anti-TREM1 Ab	
CASP1	
Casp1-Casp5	
CASP5	
CCL2	8
CCL3	
CCL7	8
CD40	4;8
CD83	
CD86	
CSF2	
CXCL3	2
DEFB4A/DEFB4B	
EBOV	
ERK1/2	5
FCGR2B	5
Flagellin	
GRB2	
ICAM1	
IL10	
IL18	
IL1B	
IL6	
IL8	7
IRAK1	
ITGA5	
ITGAX	
ITGB1	
JAK2	
L-Ala-?-D-Glu-meso-diaminopimelinic acid	
LAT2	
lipopolysaccharide	
lipoteichoic acid	
MARV	
MARV GP	
MPO	2;6
MYD88	4
N-acetylmuramyl-L-alanyl-D-isoglutamine	
NFkB (complex)	
NLR	
NOD2	
Pam3-Cys	
PLC gamma	5
poly rI:rC-RNA	
prostaglandin E2	
resiquimod	
SIGIRR	
ST2	
STAT3	
STAT5a/b	4
Tlr	4;8;3*
TLR2	4
TLR4	4
TNF	
TREM1	
TYROBP	3

Genes involved in TREM1 signaling canonical pathway according to Ingenuity Integrated Pathway analysis. Genes identified in our 409 gene total geneset are indicated in the right column. * TLR genes include TLR2,4,5,6,7,8 and were identified by multiple studies, all genes identified count in overlap with pathway count according to Ingenuity

A soluble variant of TREM1 has also been identified (sTREM1). This results from the shedding of cell surface TREM1 by metalloproteinases, and is thought to negatively regulate TREM receptor signaling through neutralization of the ligands [35]. In patients with pulmonary TB sTREM1 has been detected in sputum [38], but levels could not discriminate pulmonary TB from community acquired pneumonia caused by other pathogens. In patients with pleural effusions, sTREM1 levels could discriminate infectious, including TB, from non-infectious causes and the levels of pleural fluid sTREM1 appeared a useful tool to discriminate Mtb infection from malignancies [39]. An independent study on pleural effusions showed a similar increase in sTREM1 levels in TB effusions compared to non-infectious effusions [40]. In addition, surface TREM1 was assessed on cells within the effusions and was undetectable in effusions from patients with TB pleuritis [40], however cell numbers and cellular composition were not described. Similarly, in BAL (bronchoalveolar lavage) cells from patients with pulmonary TB no significant increase in cell-surface TREM1 expression was observed compared to BAL cells from non-infectious controls, whereas BAL cells from patients with other pulmonary infections did express increased TREM1 levels on macrophages and neutrophils [41]. Thus, TREM1 related signaling seems important during active TB disease; although cellular TREM1 expression levels may not be changed, levels of soluble sTREM1 are increased at the site of disease.

#### Myeloid lineage cells

In addition to TREM1 signaling, a number of other pathways associated with myeloid cell function or activation were prominently identified in the Ingenuity analysis (Table 3). These included: ‘Fcγ receptor-mediated phagocytosis in macrophages and monocytes’; ‘role of pattern recognition receptors in recognition of bacteria and viruses’; ‘role of macrophages, fibroblasts and endothelial cells in Rheumatoid Arthritis’; ‘Dendritic cell maturation’; Production of Nitric Oxide and reactive oxygen species in macrophages'; ‘Toll-like receptor signaling'; ‘IL-12 signaling and production in macrophages’. In Table 3 these are grouped together into a ‘myeloid’ category, which was then applied to allocate individual genes into functional categories in Table S1. Increased expression of genes associated with Toll-like receptor signaling [18], [19], [26] and Fcγ Receptor signaling [26], [29] were previously reported in patients with active TB disease. Grouping of genes according to the canonical pathway based groups resulted in categorization of 107 out of the total 409 genes in the dataset. The myeloid signature thus identified is strong and also more clearly represented than in any of the individual studies: 56/107 (52.3%) genes that were grouped into canonical pathways were in the myeloid category, although the majority overlaps with other categories as well, including TREM1 signaling.

As with the Ingenuity based pathway analysis, most of the GSEA gene sets we identified represented a myeloid biased gene signature and an inflammatory immune response. The gene sets SEKI_INFLAMMATORY_RESPONSE_LPS_UP, ICHIBA_GRAFT_VERSUS_HOST_DISEASE_35D_UPICHIBA_GRAFT_VERSUS_HOST_DISEASE_D7_UP, JISON_SICKLE_CELL_DISEASE_UP and REACTOME_IMMUNE_SYSTEM overlap with each other and comprise many genes associated with myeloid cells (Table 4). The dominance of these gene signatures reflect the findings of the studies the signatures were derived from. Cliff et al [31], Berry et al [17] and Ottenhoff et al [32] all highlight an increased myeloid cell inflammatory responses in active TB patients when compared to controls.

Network analysis revealed a strong contribution of genes associated with myeloid cells in the most dominant network (figure 1), however many genes overlapped with more than one process (Table S1, Figure 1). These comprised signaling pathways around Akt, MYD88 and the NFκB complex as central players in the network, indicating an important role for inflammation, phagocytes and professional antigen presenting cells including macrophages. Indeed the early microarray studies performed in TB patients indicated that genes with roles in inflammation and immunity were most abundantly expressed [18], [20], [26], [32] and most inflammation related genes differentially expressed were derived from monocytes [17], [19], [29], [32]. Interestingly, Cliff et al. speculated that some of the genes specifically upregulated during TB disease can be expressed by macrophages and DCs but not by monocytes, and thus may reflect activated APCs, possibly cells trafficking between lungs and lymphoid tissue via the blood [31]. This could also explain the relatively high myeloid gene expression observed in TB patients and in particular the involvement of TLR and Fc receptor induced signaling, since these processes would normally be expected to occur at the site of disease rather than in the circulation. The pathways associated with these myeloid cells point towards a strong role for direct pathogen related processes, even in the circulation (not the disease site in TB).

#### T-cells and B-cells

Although the emerged profiles are dominated by inflammatory and myeloid gene signatures there was one gene set associated with CD4 T Cell/follicular helper T cell activation (PICCALUGA_ANGIOIMMUNOBLASTIC_LYMPHOMA_UP) [42] (Table 4). CD4 T cells are important for the control of Mtb infection and TB disease, proliferate and are activated in response to antigens from M. tuberculosis. It is possible that these follicular helper T cells are contributing to the formation of B cell follicles during active TB disease. Studies of human TB granulomas have identified B cell follicle structures which may contribute to the immune response in TB, and activated B cells have been found in granulomas of nonhuman primates infected with Mtb [43], [44].

As expected also canonical pathways associated with B cell function (as previously reported by [19], [31]), T cell function (previously reported by [31]) and more general inflammation processes were identified by Ingenuity analysis (Table 3). The potential functional implications of B cells in TB disease have been debated for decades, in particular because intracellular pathogens are considered to be sequestered from circulating antibodies. However, functional significance of B cells in TB disease has been demonstrated in mouse models: B cell deficient mice appear more susceptible to TB [45], and Fc-receptors play a role in protection. Furthermore, in addition to the presence of B cells in human TB lesions as described above, the expression of human FcγR1 is a consistent and strong component in TB biomarker signatures [19], [29], [30]. These observations suggest that B cells may play a hitherto unappreciated role in immunity in TB. This is further supported by recent results that suggest that also intracellular binding of antibody to pathogens can take place via a cytosolic Fc-receptor called TRIM21 [46], [47]. Pathogen bound antibody triggering of TRIM21 subsequently stimulated transcription factor pathways including NF-kB, IRF7 and others, resulting in immune activation and inflammatory signals. This may open up an interesting new angle for the potential role of B cells and in particular antibodies in the combat against TB, which should be explored in more detail. Interestingly, our network based analysis of genes expressed in peripheral blood from TB patients does include TRIM21 (Figure 1). Gene expression of TRIM21 was detected in cohorts of TB patients compared to healthy controls [19] (Table S1) and was grouped close to Fc Receptors and its defined signaling molecule IRF7 in the Ingenuity network analysis.

#### Hematopoiesis

A process which was not expected but clearly represented in the canonical pathway analysis was hematopoiesis, represented by ‘Erythropoietin signaling’; ‘IL-3 signaling’; ‘FLT3 signaling in hematopoietic progenitor cells’; ‘prolactin signaling’ and ‘HGF signaling’. This indicates that in the blood of patients with TB disease active remodeling apparently is ongoing, either with renewal of hematopoietic cells at a possibly increased rate compared to non-TB controls or with cells emerging from the bone marrow and on their way to the site of inflammation (mostly the lung in TB patients). Recently it has been demonstrated that *Mtb* may hijack mesenchymal stem cells and may survive in the CD271+ stem cells for a long period after successful treatment of pulmonary TB, hiding from the immune system [48]. This suggests that stem cells in the bone marrow may be active players in TB disease, revealing a new cell type involved in TB pathogenesis. Another interesting link between mycobacterial infections and stem cells is the recent observation that *Mycobacterium leprae* is capable of reprogramming host gene expression in adult Schwann cells and induces a dedifferentiation program towards stem cell like cells (SLC) [49]. These SLC can subsequently redifferentiate into end stage tissue cells, and also release bacteria onto macrophages locally in tissues [49]. It remains unknown whether *Mtb* also possesses the capacity to reprogram host cells by activating pathways characteristic for hematopoiesis, but this may be an interesting possibility that fits with the observed changes in gene expression profiles. Interestingly, the vast majority of genes categorized as ‘hematopoiesis’ is also associated with several myeloid functions (Table 3).

#### Type I Interferon Signaling

Berry et al [17] were the first to describe that Type I interferon signaling was increased in TB patients. The pathway MOSERLE_IFNA_RESPONSE was amongst the 15 most significant pathways identified by GSEA in active TB disease (Table 4). Surprisingly, interferon α/β related signaling pathways were not amongst the top pathways represented by our dataset in Ingenuity (Table 3), despite it being detected with high significance in several individual studies. Interferon signaling reached statistical significance (p = 0.000026), but was lost during application of gating criteria, due to the small number of genes in the pathway (<50) and the small number of genes overlapping with our dataset (<10). However, because of the interest in IFNα/β signaling in TB disease, we added a category IFNα/β to table 3 based on all 3 canonical pathway datasets related to type I interferon signaling in Ingenuity ‘interferon signaling’; ‘role of JAK1, JAK2 and TYK2 in interferon signaling’ and ‘role of PKR in interferon induction and antiviral response’. IFN signaling represented 13/107 (12.1%) genes in our dataset and was thereby not the most dominant pathway (figure 1, Table S1). Signaling through IFNα/β seems an important pathway associated with TB disease in most independent studies, although many molecules involved in downstream IFNα/β pathways are not uniquely involved in transmitting signals derived from IFNα/β receptors but may also be critical components in other pathways.

Type I interferons are classically known as early response molecules in the context of viral infections and are well known immune modulators. Type I interferon signaling in mycobacterial infections, as shown in progressive leprosy disease, may contribute to disease pathogenesis or protection, depending on the balance between IFNα/β and IFNγ [50], in which IFNα/β may actively inhibit IFNγ signalling. Interestingly in virus infection models the IFNα/β response mediated enhanced antiviral activity during acute infection but exerted strong immunomodulatory effects during chronic infection [51]. Both TB and leprosy are long term, chronic infections; in both diseases an increased expression of IFNβ induced genes has been observed, suggesting its potential involvement in tissue damage and inflammation, and in regulating adaptive IFNγ responses. It may be speculated that patients with latent Mtb infection have strong IFNγ induced responses which are downregulated by IFNα/β during reactivation from latent to active TB disease [4], [50].

Macrophages or monocytes infected with Mtb produce and secrete type I interferons [52]. Mtb strains with increased virulence induce increased IFNA mRNA in lungs of infected mice [53], and lower local levels of pro-inflammatory cytokine mRNAs (IL-6, IL-12, TNFα and IFNγ). The effect of IFNα/β was further investigated by administration of IFNα to mice following infection with Mtb, resulting in increased pulmonary bacterial loads and reduced survival of the mice [53]. In vitro, addition of type I interferons to BCG infected macrophages resulted in increased outgrowth of BCG, suggesting direct effects of type I interferons on infected cells in favor of the mycobacteria [54]. The increase in type I interferons and IFNα/β signaling during active TB disease may therefore reflect IFNα/β production by infected monocytes/macrophages and facilitate and maintain rather than clear chronic infection.

#### Fibrosis

Statistically, using GSEA analysis, the greatest overlap of our gene-set was observed with the gene set MARKEY_RB1_ACUTE_LOF_UP, a gene set expressed in fibroblasts [55] (Table 4). Fibroblasts secrete extracellular matrix molecules, while extracellular matrix destruction is necessary for the growth and persistence of Mtb: Mtb is a potent inducer of the metalloproteinases (MMPs) which destroy extracellular matrix [56]. MMP9 and MMP25 are found among our gene list of 409. MMP expression is enhanced by G protein coupled receptor signalling and type 1 interferon – both of which are represented in our GSEA analysis (REACTOME_GPCR_LIGAND_BINDING and MOSERLE_IFNA_RESPONSE). MMP9 is the most abundant of the MMPs and has been found to correlate with disease severity in TB patients [57]. The neutrophil specific matrix metalloproteinase MMP25 degrades substrates found in fibroblasts and may enhance the phagocytic removal of neutrophils from inflammatory sites [58].

## Conclusions

Analysis of gene-expression data is a complex process which can be performed using many different strategies. Commonly genes are grouped according to similar changes in expression profiles, e.g. using non-hierarchical clustering. More recently, groups of genes have been clustered into modules based on their shared expression (and likely involvement) in biological processes [33]. These modular representations have become more popular in annotating function to gene expression data. However, joint expression of genes during certain processes/diseases does not necessarily demonstrate functional relatedness. Related gene expression events may be dynamic and occur in sequence rather than simultaneously such that genes with strong functional relations may not necessarily be detected at the same time point in the (disease) process and therefore may not group into the same cluster or module. The assessment of pathways and processes that seem key in the disease process seems to be more valuable than merely assessing the combination of genes with the highest changes in expression levels.

**Figure 4:**
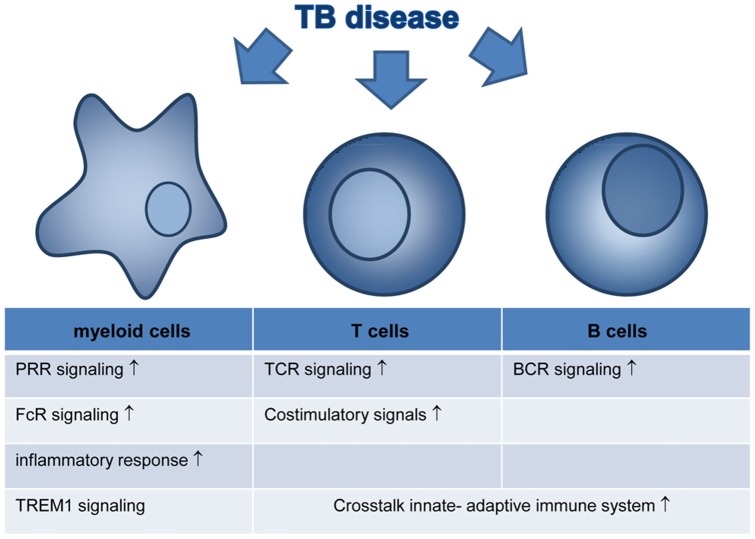
Schematic representation of events during active TB Disease. Pathway and process based analysis suggests that these processes are key players in TB disease pathogenesis.

Here we explored functional interactions based on known molecular interactions (Ingenuity) and enrichment of genes in biological pathways (GSEA) to analyze gene expression data described in TB disease at a higher aggregation level, from an helicopter perspective. Dominant pathways may not only serve as biomarkers at the transcription level as such, but products from these pathways or cellular responses induced by that particular pathway may also be significant indicators of disease, potentially allowing easier assessment of pathway activity in simpler tests. Future assessments of biomarkers for vaccine-induced protection may benefit from similar, pathway/process based analyses to allow more powerful biomarker selection (Figure 4).

Interestingly, multiple genes affected in TB disease were involved in more than a single pathway (Table S1, Figure 1), emphasizing the impact of individual signaling components in multiple pathways. Therefore, assessment of total pathway function or at least multiple components from the same pathways may be a more reliable measurement of such pathways than individual markers that are functionally involved in many different signal transduction chains.

The combined analysis of TB biomarkers of disease identified by 8 independent studies revealed a less dominant role for the IFNα/β related genes, but revealed a very strong involvement of myeloid cells. In particular signaling through Pattern Recognition Receptors, Fc receptors, fibrosis and TREM1 seemed key players during active TB disease. These findings may be somewhat unexpected, mostly because all profiling was performed in whole blood or purified PBMCs and not at the site of disease. In particular in Mtb infection, the local activation of myeloid cells and receptors involved in pathogen recognition are expected to be key players in the disease process, yet also systemic activation of these pathways is emphasized by our analysis. These cells may either traffic from the site of disease into the circulation or may have been indirectly activated by pathogen derived products that have entered the circulation, thus mirroring activated phagocytes at the site of disease. It is somewhat surprising to detect a prominent activated myeloid cell signature in the circulation, because generally activation would occur in tissues and cells would subsequently follow the lymphoid system to migrate towards lymphoid organs. Apparently, during active TB disease inflammation may be of such magnitude that the inflammatory site spills over into the circulation such that activated myeloid cells can be detected in peripheral blood (Figure 4).

In this context, the identification of TREM1 signalling is interesting, providing a new angle for activation of monocytic cells by *Mtb*. Potentially, *Mtb* derived molecules target this pathway, in synergy with TLRs, to activate innate and adaptive immune responses. Additional new leads may include pathways associated with hematopoiesis and B cell activation. Several pathways associated with hematopoiesis were identified by Ingenuity pathway analysis, which may support new recent leads of circulating hematopoietic progenitor cells during TB disease. However, the individual genes that fit into these pathways are also involved in myeloid cell activation and inflammation. Therefore genes exclusively associated with hematopoiesis in particular need to be investigated to determine the relevance of hematopoiesis in TB disease. B cell activation in the circulation of patients with TB disease may also require more detailed investigation, in particular since also TB granulomas harbour B cells suggesting active involvement with disease.

All of these pathways deserve more in-depth (functional) analysis in TB disease and hopefully will guide exploration of new therapeutic targets for TB disease.

Thus, our ‘helicopter’ like pathway-based analysis of multiple independent studies reveals novel insights in the pathogenesis and potential biomarkers of TB disease, implying a strong role for myeloid cells in TB pathogenesis which deserves more in-depth investigation.

## Supporting Information

Table S1 **All genes identified by the 8 independent global genome-wide gene expression analyses.** Genes names are the official gene names according to gene cards (www.genecards.org) and alternative names are given in the second column. Manuscript numbers refer to Table 2. Classification into modules, functional groups according to Ingenuity and GSEA was performed according to Tables 3 & 4.
(XLS)
